# Delayed Recurrence of Atypical Pulmonary Carcinoid Cluster: A Rare Occurrence

**DOI:** 10.1155/2014/620814

**Published:** 2014-11-18

**Authors:** Salim Surani, Jennifer Tan, Alexandra Ahumada, Saherish S. Surani, Sivakumar Sudhakaran, Joseph Varon

**Affiliations:** ^1^Pulmonary, Critical Care & Sleep Medicine, Texas A&M University, Corpus Christi, 1177 West Wheeler Avenue, Suite 1, Aransas Pass, TX 78336, USA; ^2^Corpus Christi Medical Center, 7101 South Padre Island Drive, Corpus Christi, TX 78412, USA; ^3^Universidad Autonoma de Baja California, Avenue Álvaro Obregón Sn, Nueva, 21100 Mexicali, BC, Mexico; ^4^Dorrington Medical Associates, 2219 Dorrington Street, Houston, TX 77030, USA; ^5^Pulmonary Associates, 1177 West Wheeler Avenue, Aransas Pass, TX 78336, USA; ^6^Texas A&M University Health Science Center, 8447 State Highway 47, Bryan, TX 77807, USA; ^7^The University of Texas Health Science Center, 7000 Fannin Street, Houston, TX 77030, USA; ^8^University General Hospital, 7501 Fannin Street, Houston, TX 77054, USA

## Abstract

Carcinoid is one of the most common tumors of the gastrointestinal tract followed by the tracheobronchial tree. Bronchial carcinoid compromises 20% of total carcinoid and accounts for 1–5% of pulmonary malignancies. Carcinoid can be typical or atypical, with atypical carcinoid compromises 10% of the carcinoid tumors. Carcinoid usually presents as peripheral lung lesion or solitary endobronchial abnormality. Rarely it can present as multiple endobronchial lesion. We hereby present a rare case of an elderly gentleman who had undergone resection of right middle and lower lobe of lung for atypical carcinoid. Seven years later he presented with cough. CT scan of chest revealed right hilar mass. Flexible bronchoscopy revealed numerous endobronchial polypoid lesions in the tracheobronchial tree. Recurrent atypical carcinoid was then confirmed on biopsy.

## 1. Introduction

The most common location of carcinoid tumors is the gastrointestinal tract, followed by the tracheobronchial tree [1]. Bronchial carcinoids are low-grade neuroendocrine malignant tumors that comprise 1–5% of all lung neoplasms [2–4]. It used to be felt as a benign tumor but when distant metastases happen, it tends to be less aggressive than the noncarcinoid lung malignancies [5–7].

In 2004, new criteria divided lung carcinoids into different groups: typical and atypical carcinoids, large cell neuroendocrine carcinoma, and small cell lung carcinoma [8]. Of the bronchial carcinoids, the typical is much more common than the atypical type (90% versus 10%) [9]. We hereby present a case of 63-year-old male who presented with recurrence of carcinoid seven years after curative resection of his carcinoid.

## 2. Case Report

63-year-old nonsmoker male presented to the office with chronic cough of 2-month duration. The cough was dry with no sputum production. Patient denies dyspnea, wheezing, hemoptysis, night sweat, and weight loss. Seven years prior to this presentation the patient had resection of right middle and lower lobe for the solitary atypical bronchoscopic biopsy proven endobronchial carcinoid just below the right minor carina. On pathology the patient's lesion confirmed atypical carcinoid. The hilar and mediastinal nodes were negative for any tumor and the margins as well were free of any tumor. Computerized tomography (CT) scan and positron emission tomography (PET) scan showed no evidence of any local or distant spread. Patient besides the previous history of carcinoid also had history of hypertension and coronary heart disease. Patient was not on any angiotensin converting enzyme inhibitor (ACE) or angiotensin receptor blocker (ARB) for the control of his blood pressure. Physical examination revealed decreased breath sound in the right lower chest. No wheezing or rhonchi were heard in both lungs.

**Figure 1:**
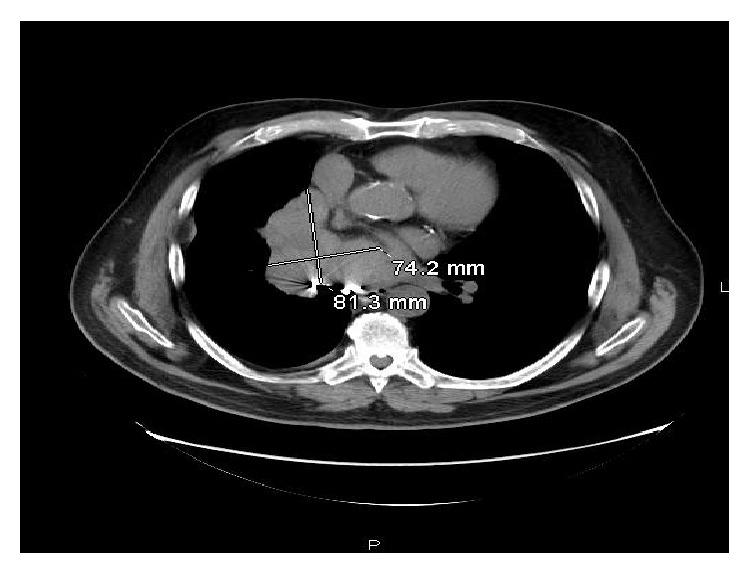
CT Scan of chest showing right hilar mass.

**Figure 2:**
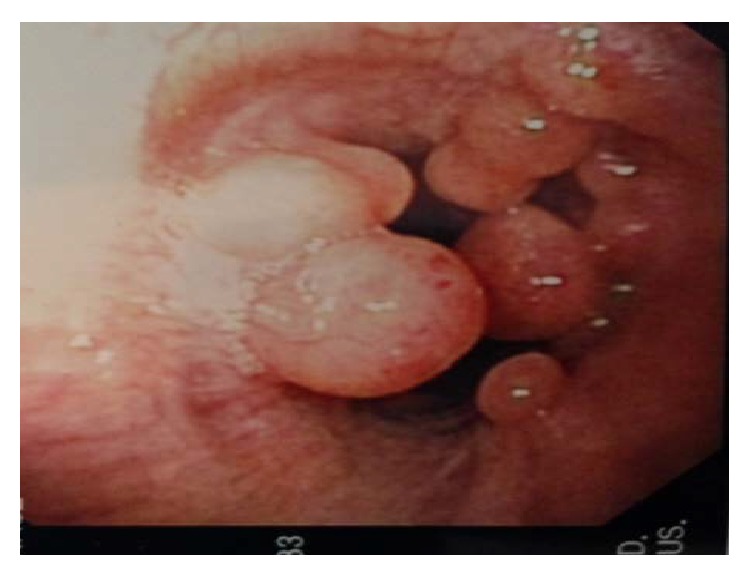
Bronchoscopic images of right main stem bronchi showing multiple polypoid lesions, which on biopsy confirmed carcinoid.

**Figure 3:**
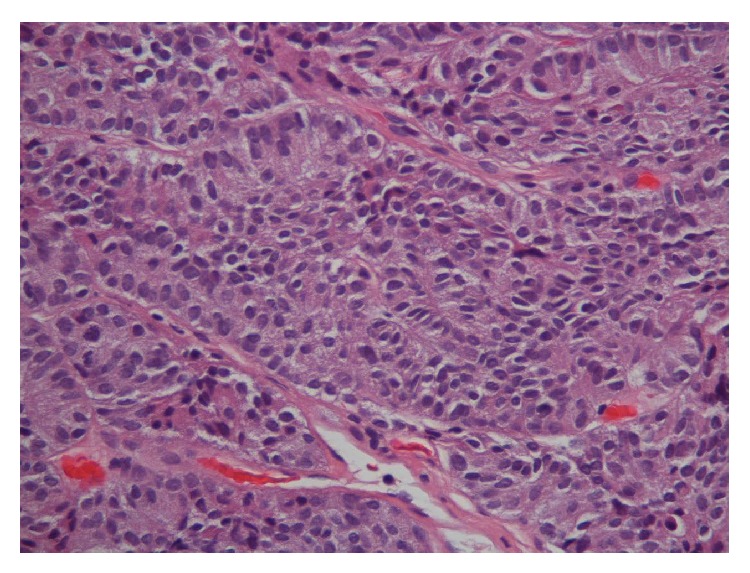
High magnification image showing well-differentiated cells forming tubular and glandular structure without atypia.

**Figure 4:**
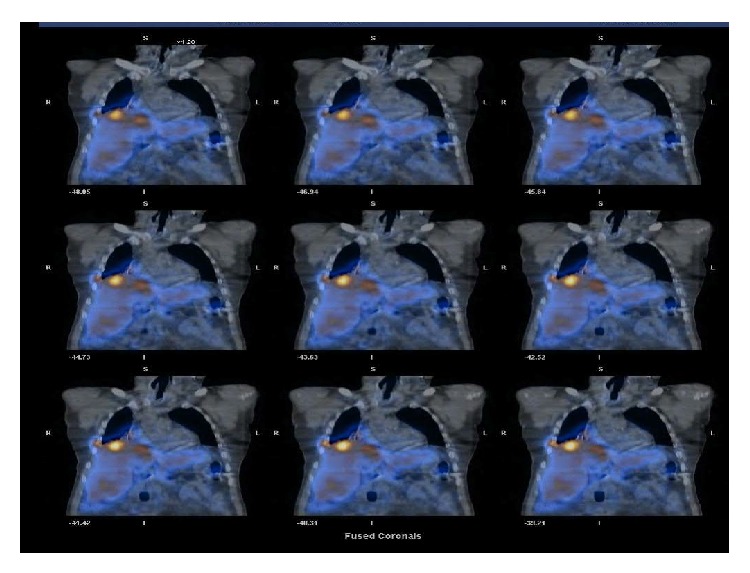
Octreotide scan showing abnormal activities in the right hilum and carinal area as well as liver suggestive of metastatic carcinoid.

In view of his chronic cough, the patient underwent X-ray of the chest, which showed some hilar fullness. A computed tomography (CT) scan of chest revealed a new 8.1 × 7.4 cm right hilar mass, with no evidence of disease noted in trachea or left lung and no evidence of any endobronchial lesions (Figure 1). PET scan showed right hilar mass that was not FDG-avid. Flexible video bronchoscopy showed numerous polypoid masses in vocal cords, trachea, and left and right mainstem bronchus (Figure 2). Biopsy of one of the lesions in the right mainstem bronchus showed a well-differentiated tumor consisting of neuroendocrine-appearing cells forming small tubular and glandular-like structures, without significant atypia or mitotic activity, consistent with carcinoid (Figure 3). Chromogranin and synaptophysin stains for neuroendocrine tumor are positive and Ki-67 stain for mitotic activity is relatively low. The patient underwent Octreotide scan which showed extensive metastatic carcinoid, including osseous metastasis, liver metastasis, right hilar, subcarinal, right pleural involvement, right chest wall, and subcutaneous tumor behind right chest (Figure 4). Patient was then started on octreotide intramuscular injections every 4 weeks. Three months after diagnosis, patient is doing well and is asymptomatic.

## 3. Discussion

Carcinoid tumors are neuroendocrine tumors that typically arise from gastrointestinal tract and the bronchus. Bronchial carcinoid tumors comprise approximately 20% of all carcinoid tumors and approximately 1–5% of all lung malignancies in adults [10, 11]. Carcinoid tumors can secrete many different types of products, the most common of which are serotonin, histamine, tachykinins, kallikrein, and prostaglandins. These products cause the classic symptoms associated with carcinoid tumors: diarrhea and cutaneous flushing [12]. Bronchial carcinoid tumors can be asymptomatic or can cause causes wheezing, dyspnea, cough, hemoptysis, and recurrent pneumonia due to bronchospasm and obstruction [13].

Due to the variability of clinical presentation of bronchial carcinoid, there can be a delay in diagnosing or even misdiagnosis. The differential diagnosis of a patient with symptoms of bronchial obstruction, bronchospasm, and hemoptysis is wide; it includes an obstructing bronchial carcinoma, endobronchial metastasis, hamartomas, aspirated foreign body, asthma, and COPD.

While the 24-hour urinary excretion of 5-hydroxyindoleacetic acid (HIAA) is a useful initial diagnostic test for carcinoid tumors arising in the midgut (jejunoileal, appendiceal, and ascending colon), it is not as helpful for carcinoid tumors arising in the foregut (gastroduodenal and bronchus) or hindgut (transverse, descending, and sigmoid colon and rectum), as they secrete less serotonin [14]. Therefore the diagnosis of bronchial carcinoid tumors sometimes can be challenging. Chest X-ray and CT scan can detect bronchial carcinoid tumors, and the diagnosis can be confirmed by bronchoscopic biopsy for central lesions and CT-guided needle biopsy for peripheral lesions. Bronchial carcinoid commonly appears as a pinkish to reddish vascular mass, attached to the bronchus by a broad base, but can have a polypoid appearance as well. While an experienced bronchoscopist can make a diagnosis based on appearance, biopsy aids in diagnosis; cytology from bronchial brushing is often not helpful [15].

There are several differences between these two groups; in atypical carcinoids, the age of presentation tends to be much higher as in our patient's case. In one study the mean was 51 years for atypical versus 43 years for typical [16, 17]. In this case, the age of presentation was 70 years old, which is higher than the average age reported in the literature. Also, in these tumors the stage is more advanced and they are larger at the time of diagnosis, which could be explained because they are located mostly in the periphery and produce fewer symptoms [7, 18]. They have been found to have a more aggressive behavior and are most likely to metastasize [19].

Bronchial carcinoids have not been found to have any associations to smoking cigarettes or exposure to tobacco smoke [16, 20].

They can present with symptoms or be asymptomatic. In several studies, 17–52% of patients presented symptoms. In our case, the patient had nonresolving cough, which was found to be the most common symptom besides hemoptysis. Other reported problems were recurrent pulmonary infection, fever, and dyspnea. Carcinoid syndrome associated with bronchial carcinoid is very rare and in most studies it was not present; only a retrospective study reported that it happened in one patient [7, 16, 20–22].

In asymptomatic patients, the disease is usually diagnosed with a simple chest X-ray, which would show a radiopaque mass [23]. Our patient underwent CT scan of the chest after abnormality was seen on the chest X-ray. The best method of diagnosis is fiberoptic bronchoscopy with biopsy, which is what our patient underwent after seeing abnormalities on the CT scan [19, 21] though the caution needs to be undertaken for any bleed, which can compromise the airway, and rigid bronchoscopy can be option too in those circumstances. In one study, mucosal infiltration and obstruction of the lumen were the two most common macroscopic features found during bronchoscopy; polypoid lesion was not as common, and it is what we found in this case [16]. Besides diagnosis, bronchoscopy can also aid in surgical planning, since it can provide spatial information for the surgeon and also could give an idea whether it is possible to resect the tumor or not [19, 24].

The management is very straightforward; surgery is the mainstay for the treatment of bronchial carcinoid, whether it is typical or atypical. Our patient did not undergo surgery as the lesion was involving trachea, vocal cords, and both mainstem bronchi. With the typical carcinoids, it is possible to remove less tissue, that is, bronchial sleeve resection, since they are not as aggressive, but, with the atypical, lobectomy and pneumonectomy are the most common options [19, 25]. For entirely intraluminal endobronchial carcinoid tumors without evidence of bronchial wall involvement or suspicious lymphadenopathy, bronchoscopic resection can be curative. Care must be taken, as potential bleeding during biopsy or resection can lead to airway compromise with flexible bronchoscopy, and for this reason rigid bronchoscopy may be preferable. Once diagnosis is established, the preferred treatment of choice for bronchial carcinoid is surgical resection, with bronchoplastic techniques (i.e., sleeve, wedge, or flap resection) in order to preserve lung parenchyma [26]. The role of adjuvant therapy of a bronchial carcinoid is a topic of controversy, as there are no prospective trials addressing the benefit of chemotherapy with or without radiation therapy for resected bronchial carcinoids. For patients with carcinoid syndrome, control of the symptoms caused by tumor's secretion of peptide and amines can be achieved by somatostatin receptor analogues such as octreotide.

The most important prognostic factor for bronchial carcinoid is the histology. As we had already discussed, typical carcinoids have a better prognosis and less rate of recurrence than atypical carcinoids [19]. In our case, the patient had atypical carcinoid and it recurred after 7 years of being asymptomatic.

The 5-year survival ranges from 89 to 92% in the typical carcinoid group and from 66.7 to 75% in the atypical carcinoid group. The 10-year survival was 82–88.9% for typical and 50–56% for atypical, as we should expect, given the more aggressive behavior in the latter group [16, 20].

However, as bronchial carcinoid tumors are a spectrum consisting of more indolent tumor to more aggressive tumors with the potential to metastasize or recur locally, long-term follow-up is needed as local or distant recurrence may occur many years after the initial diagnosis and treatment as in this case [7]. There is not a consensus on the optimal surveillance strategy after treatment. Some proposed strategies include high-resolution CT (with or without flexible bronchoscopy) annually for the more indolent “typical” bronchial carcinoids; for the more aggressive “atypical” bronchial carcinoids, it is every 6 months for the first 2 years and then annually [27].

## 4. Conclusion

The most important prognostic factor for bronchial carcinoid is the histology. As we had already discussed, typical carcinoids have a better prognosis and less rate of recurrence than atypical carcinoids [19]. In our case, the patient had atypical carcinoid and it recurred after 7 years. The 5-year survival ranges from 89 to 92% in the typical carcinoid group and from 66.7 to 75% in the atypical carcinoid group. The 10-year survival was 82–88.9% for typical and 50–56% for atypical, as we should expect, given the more aggressive behavior in the latter group [16, 20]. It is important to recognize that the carcinoid can recur after curative resection after several years. Clinical vigilance is necessary especially in the patients with atypical carcinoid.
